# 5′,11′-Dihydro­dispiro­[cyclo­hexane-1,6′-indolo[3,2-*b*]carbazole-12′,1′′-cyclo­hexa­ne]

**DOI:** 10.1107/S1600536811051208

**Published:** 2011-12-03

**Authors:** Ilia A. Guzei, Lara C. Spencer, Eric Codner, Joshua M. Boehm

**Affiliations:** a Department of Chemistry, University of Wisconsin–Madison, 1101 University Ave, Madison, WI 53706, USA; b Department of Chemical and Biological Engineering, University of Wisconsin–Madison, 1415 Engineering Drive, Madison, WI 53706, USA

## Abstract

The title compound, C_28_H_30_N_2_, is a symmetrical 2:2 product from the condensation of indole and cyclo­hexa­none. It is the only reported 5,11-dihydro­indolo[3,2-*b*]carbazole compound in which the spiro atoms are quaternary C atoms. Crystals were grown by vapor diffusion in a three-zone electric furnace. The mol­ecule resides on a crystallographic inversion center. The cyclo­hexyl rings are in a slightly distorted chair conformation, whereas the indole units and the spiro-carbons are coplanar within 0.014 Å.

## Related literature

For condensations of indole with cyclo­hexa­none that yield 1:1 or 1:2 products, see: Yadav *et al.* (2001 ▶). For indole–ketone condensation by forming vinyl­indole followed by a Diels–Alder reaction, see: Noland *et al.* (1993 ▶). Recrystallization by the vapor-phase diffusion approach is explained in Kloc *et al.* (1997 ▶). For information on the related compound *trans*-6,12-diphenyl-5,6,11,12-tetra­hydro­indolo[3,2-*b*]carbazole dimethyl sulfoxide tetra­hydro­furan solvate, see: Gu *et al.* (2009 ▶). Related compounds were found in the Cambridge Structural Database (Allen, 2002 ▶). Geometrical parameters were analyzed using *Mogul* (Bruno *et al.*, 2002 ▶).
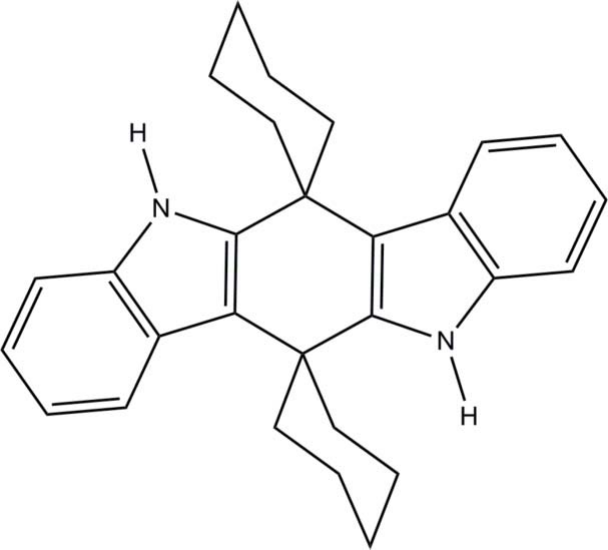


## Experimental

### 

#### Crystal data

C_28_H_30_N_2_
*M* *_r_* = 394.54
Monoclinic, 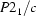

*a* = 7.4655 (2) Å
*b* = 13.6820 (4) Å
*c* = 10.5348 (3) Å
β = 109.380 (1)°
*V* = 1015.08 (5) Å^3^
*Z* = 2
Cu *K*α radiation
μ = 0.57 mm^−1^
*T* = 100 K
0.38 × 0.30 × 0.19 mm

#### Data collection

Bruker SMART APEXII area-detector diffractometer
Absorption correction: analytical (*SADABS*; Bruker, 2007 ▶) *T*_min_ = 0.813, *T*_max_ = 0.900
20619 measured reflections
1826 independent reflections
1779 reflections with *I* > 2σ(*I*)
*R*_int_ = 0.022

#### Refinement

*R*[*F* ^2^ > 2σ(*F* ^2^)] = 0.041
*wR*(*F* ^2^) = 0.113
*S* = 0.99
1826 reflections
140 parameters
H atoms treated by a mixture of independent and constrained refinement
Δρ_max_ = 0.35 e Å^−3^
Δρ_min_ = −0.22 e Å^−3^

### 

Data collection: *APEX2* (Bruker, 2007 ▶); cell refinement: *SAINT-Plus* (Bruker, 2007 ▶); data reduction: *SAINT-Plus*; program(s) used to solve structure: *SHELXTL* (Sheldrick, 2008 ▶); program(s) used to refine structure: *SHELXTL*, *FCF_filter* (Guzei, 2007 ▶) and *INSerter* (Guzei, 2007 ▶); molecular graphics: *SHELXTL* and *DIAMOND* (Brandenburg, 1999 ▶); software used to prepare material for publication: *SHELXTL*, *publCIF* (Westrip, 2010 ▶) and modiCIFer (Guzei, 2007 ▶).

## Supplementary Material

Crystal structure: contains datablock(s) global, I. DOI: 10.1107/S1600536811051208/nk2127sup1.cif

Structure factors: contains datablock(s) I. DOI: 10.1107/S1600536811051208/nk2127Isup2.hkl

Additional supplementary materials:  crystallographic information; 3D view; checkCIF report

## Figures and Tables

**Table:** 

N1—C1	1.3743 (16)
N1—C8	1.3872 (16)

**Table:** 

C1—N1—C8	109.12 (11)

**Fig. 1.**
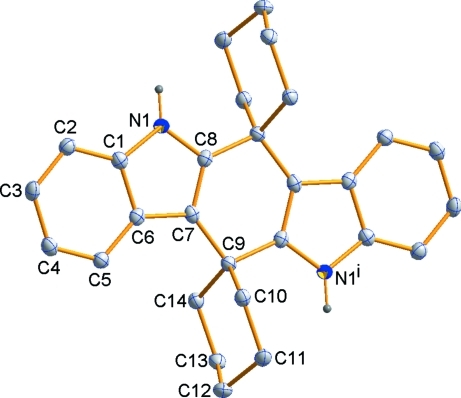
Molecular structure of (I) (Brandenburg, 1999). Displacement ellipsoids are shown at the 50% probability level and C-bound H atoms are omitted. The compound resides on a crystallographic inversion center. Symmetry transformations used to generate equivalent atoms: (i) -x + 1,-y,-z.

## supplementary crystallographic information

### Comment

Condensations of indole with cyclohexanone yield a variety of products, often 1:1 or 1:2 structures in which the carbonyl carbon is attached to the 3-position of one or more indole moieties (Yadav *et al.*, 2001). These products are often useful as pharmaceutical intermediates, and a variety of catalysts and reaction conditions have been explored. A particularly interesting example of indole-ketone condensation chemistry is vinylindole formation followed by the Diels-Alder reaction (Noland *et al.*, 1993). While investigating this reaction we isolated highly symmetrical 2:2 condensation products from preparations containing indole and a cyclic ketone (cyclohexanone, *tert*-butyl cyclohexanone, or cyclopentanone) using hydrochloric acid as a catalyst. These 2:2 condensation products possess unusual physical properties for this class of compound, including limited solubility in most solvents, decomposition without melting at temperatures over 300 °C, and fluorescence despite the absence of extended intramolecular conjugation. Due to the lack of a suitable recrystallization solvent we employed the vapor-phase diffusion approach of Kloc *et al.* (1997) to produce crystals of dispiro[cyclohexane-1,6'(1'*H*)-indolo[3,2-*b*]carbazole-12'(1*H*),1''-cyclohexane] (I).

Data mining of the Cambridge Structural Database (CSD; November 2011 update; Allen, 2002) revealed that (I) is the only crystallographically characterized condensation product of indole and cyclohexanone in which the spiro atoms are quaternary carbon atoms.

The molecule of (I) resides on a crystallographic inversion center. The amino hydrogen atoms do not participate in hydrogen bonding interactions due to the lack of acceptors. In contrast, the related compound *trans*-6,12-diphenyl-5,6,11,12-tetrahydroindolo[3,2-*b*]carbazole dimethyl sulfoxide tetrahydrofuran solvate (II) which also has hydrogen atoms on nitrogen atoms forms hydrogen bonding interactions with the oxygen atoms of the dimethyl sulfoxide solvent molecules (Gu *et al.*, 2009).

A *Mogul* (Bruno *et al.*, 2002) structural check confirmed that the geometrical parameters of (I) are typical except for the C10—C9—C14 angle. The latter measures 111.20 (10)° and is more obtuse than the average angle of 108.3 (9)° computed for related compounds. The difference is statistically significant. The two cyclohexyl substituents most closely resemble a chair conformation. The 5,11-dihydroindolo[3,2-*b*]carbazole core is planar within 0.0142 Å.

### Experimental

Indole (3 g) was dissolved in 25 ml of cyclohexanone. Approximately 0.25 ml of concentrated HCl was added and the mixture was stirred at room temperature for 7–14 days. The resulting pink-white precipitate was isolated using vacuum filtration and washed in refluxing acetonitrile for 60 min.

In an alternative preparation, indole (3 g) and cyclohexanone (2.5 g) were dissolved in 25 ml of acetonitrile. Approximately 0.1 ml of concentrated HCl was added and the mixture was heated to reflux for 24 h. The product was recovered from an accompanying intractable tarry material by washing with acetone followed by reflux in fresh acetonitrile.

Crystals were grown in a three-zone electric furnace. A 1 g sample of the material was placed on a microscope cover slip and inserted into a 25 mm quartz tube. The tube was placed in the furnace and connected to a supply of argon. The argon flow was adjusted to 2 ml/min, the tube was purged of air and heated in three zones. The first zone contained the initial sample and was heated to 308–310 °C to promote volatilization. The second zone was the region of molecular transport and was heated to 280–290 °C. The third zone was heated to 200 °C to encourage crystal deposition. These heating and gas flow conditions yielded needle-shaped crystals approximately 500 µm in the short dimensions and 10–15 mm long over 14 h.

### Refinement

All H-atoms attached to carbon atoms were placed in idealized locations and refined as riding with appropriate thermal displacement coefficients *U*ĩso(H) = 1.2 times *U*eq(bearing atom). Default effective *X*—H distances for T = -173.0°C C(*sp*^3^)–2H=0.99, C(*sp*^2^)–H=0.95. The hydrogen atom attached to N1 was located in the difference map and refined independently.

### Crystal data

**Table:**

C_28_H_30_N_2_	*F*(000) = 424
*M**_r_* = 394.54	*D*_x_ = 1.291 Mg m^−^^3^
Monoclinic, *P*2_1_/*c*	Cu *K*α radiation, λ = 1.54178 Å
Hall symbol: -P 2ybc	Cell parameters from 9920 reflections
*a* = 7.4655 (2) Å	θ = 5.5–67.3°
*b* = 13.6820 (4) Å	µ = 0.57 mm^−^^1^
*c* = 10.5348 (3) Å	*T* = 100 K
β = 109.380 (1)°	Block, colourless
*V* = 1015.08 (5) Å^3^	0.38 × 0.30 × 0.19 mm
*Z* = 2	

### Data collection

**Table:**

Bruker SMART APEXII area-detector diffractometer	1826 independent reflections
Radiation source: fine-focus sealed tube	1779 reflections with *I* > 2σ(*I*)
graphite	*R*_int_ = 0.022
0.50° ω and 0.5 ° φ scans	θ_max_ = 67.7°, θ_min_ = 5.5°
Absorption correction: analytical (*SADABS*; Bruker, 2007)	*h* = −8→8
*T*_min_ = 0.813, *T*_max_ = 0.900	*k* = −16→16
20619 measured reflections	*l* = −12→12

### Refinement

**Table:**

Refinement on *F*^2^	Primary atom site location: structure-invariant direct methods
Least-squares matrix: full	Secondary atom site location: difference Fourier map
*R*[*F*^2^ > 2σ(*F*^2^)] = 0.041	Hydrogen site location: inferred from neighbouring sites
*wR*(*F*^2^) = 0.113	H atoms treated by a mixture of independent and constrained refinement
*S* = 0.99	*w* = 1/[σ^2^(*F*_o_^2^) + (0.0708*P*)^2^ + 0.5526*P*] where *P* = (*F*_o_^2^ + 2*F*_c_^2^)/3
1826 reflections	(Δ/σ)_max_ < 0.001
140 parameters	Δρ_max_ = 0.35 e Å^−^^3^
0 restraints	Δρ_min_ = −0.22 e Å^−^^3^

### Special details

**Table:**

Geometry. All e.s.d.'s (except the e.s.d. in the dihedral angle between two l.s. planes) are estimated using the full covariance matrix. The cell e.s.d.'s are taken into account individually in the estimation of e.s.d.'s in distances, angles and torsion angles; correlations between e.s.d.'s in cell parameters are only used when they are defined by crystal symmetry. An approximate (isotropic) treatment of cell e.s.d.'s is used for estimating e.s.d.'s involving l.s. planes.
Refinement. Refinement of *F*^2^ against ALL reflections. The weighted *R*-factor *wR* and goodness of fit *S* are based on *F*^2^, conventional *R*-factors *R* are based on *F*, with *F* set to zero for negative *F*^2^. The threshold expression of *F*^2^ > σ(*F*^2^) is used only for calculating *R*-factors(gt) *etc*. and is not relevant to the choice of reflections for refinement. *R*-factors based on *F*^2^ are statistically about twice as large as those based on *F*, and *R*- factors based on ALL data will be even larger.

### Fractional atomic coordinates and isotropic or equivalent isotropic displacement parameters (Å^2^)

**Table:**

	*x*	*y*	*z*	*U*_iso_*/*U*_eq_	
N1	0.79965 (15)	0.12731 (8)	0.03500 (11)	0.0165 (3)	
H1	0.837 (2)	0.1597 (11)	−0.0234 (16)	0.017 (4)*	
C1	0.87673 (18)	0.13818 (9)	0.17213 (13)	0.0164 (3)	
C2	1.02992 (18)	0.19577 (9)	0.24794 (13)	0.0182 (3)	
H2	1.0995	0.2347	0.2059	0.022*	
C3	1.07633 (18)	0.19384 (9)	0.38671 (13)	0.0191 (3)	
H3	1.1790	0.2324	0.4410	0.023*	
C4	0.97353 (19)	0.13558 (9)	0.44782 (13)	0.0190 (3)	
H4	1.0083	0.1353	0.5431	0.023*	
C5	0.82312 (18)	0.07871 (9)	0.37278 (13)	0.0173 (3)	
H5	0.7556	0.0394	0.4160	0.021*	
C6	0.77079 (18)	0.07954 (9)	0.23203 (13)	0.0157 (3)	
C7	0.62336 (17)	0.03331 (9)	0.12319 (12)	0.0154 (3)	
C8	0.64558 (17)	0.06437 (9)	0.00578 (13)	0.0151 (3)	
C9	0.47366 (17)	−0.03714 (9)	0.13577 (12)	0.0153 (3)	
C10	0.57699 (18)	−0.12878 (9)	0.21510 (13)	0.0170 (3)	
H10A	0.6210	−0.1696	0.1537	0.020*	
H10B	0.6910	−0.1065	0.2886	0.020*	
C11	0.45854 (19)	−0.19262 (9)	0.27583 (13)	0.0189 (3)	
H11A	0.3571	−0.2254	0.2027	0.023*	
H11B	0.5403	−0.2438	0.3329	0.023*	
C12	0.3696 (2)	−0.13171 (10)	0.36026 (13)	0.0217 (3)	
H12A	0.4709	−0.1009	0.4354	0.026*	
H12B	0.2941	−0.1746	0.3990	0.026*	
C13	0.24144 (19)	−0.05256 (10)	0.27410 (13)	0.0194 (3)	
H13A	0.1855	−0.0135	0.3306	0.023*	
H13B	0.1362	−0.0837	0.2020	0.023*	
C14	0.35216 (18)	0.01490 (9)	0.21105 (13)	0.0172 (3)	
H14A	0.4375	0.0562	0.2831	0.021*	
H14B	0.2607	0.0589	0.1468	0.021*	

### Atomic displacement parameters (Å^2^)

**Table:**

	*U*^11^	*U*^22^	*U*^33^	*U*^12^	*U*^13^	*U*^23^
N1	0.0180 (6)	0.0177 (6)	0.0138 (6)	−0.0033 (4)	0.0054 (4)	0.0008 (4)
C1	0.0180 (6)	0.0154 (6)	0.0148 (6)	0.0033 (5)	0.0041 (5)	0.0004 (5)
C2	0.0191 (7)	0.0160 (6)	0.0192 (7)	−0.0008 (5)	0.0058 (5)	0.0009 (5)
C3	0.0178 (6)	0.0174 (6)	0.0181 (7)	−0.0022 (5)	0.0007 (5)	−0.0022 (5)
C4	0.0214 (7)	0.0188 (7)	0.0149 (6)	0.0021 (5)	0.0036 (5)	−0.0005 (5)
C5	0.0196 (6)	0.0158 (6)	0.0166 (6)	0.0006 (5)	0.0063 (5)	0.0002 (5)
C6	0.0160 (6)	0.0130 (6)	0.0183 (6)	0.0021 (5)	0.0061 (5)	−0.0006 (5)
C7	0.0156 (6)	0.0147 (6)	0.0144 (6)	0.0027 (5)	0.0032 (5)	−0.0015 (5)
C8	0.0140 (6)	0.0133 (6)	0.0177 (6)	0.0008 (4)	0.0050 (5)	−0.0007 (5)
C9	0.0171 (6)	0.0151 (6)	0.0138 (6)	0.0007 (5)	0.0055 (5)	0.0003 (5)
C10	0.0178 (6)	0.0167 (7)	0.0152 (6)	0.0004 (5)	0.0039 (5)	−0.0008 (5)
C11	0.0223 (7)	0.0159 (6)	0.0159 (6)	−0.0017 (5)	0.0030 (5)	0.0019 (5)
C12	0.0276 (7)	0.0224 (7)	0.0169 (6)	−0.0049 (5)	0.0099 (6)	0.0016 (5)
C13	0.0204 (7)	0.0224 (7)	0.0176 (6)	−0.0027 (5)	0.0090 (5)	−0.0032 (5)
C14	0.0191 (6)	0.0169 (6)	0.0158 (6)	−0.0002 (5)	0.0060 (5)	−0.0013 (5)

### Geometric parameters (Å, °)

**Table:**

N1—C1	1.3743 (16)	C9—C8^i^	1.5080 (17)
N1—C8	1.3872 (16)	C9—C14	1.5608 (16)
N1—H1	0.876 (17)	C9—C10	1.5612 (17)
C1—C2	1.4001 (18)	C10—C11	1.5264 (17)
C1—C6	1.4137 (18)	C10—H10A	0.9900
C2—C3	1.3862 (19)	C10—H10B	0.9900
C2—H2	0.9500	C11—C12	1.5227 (19)
C3—C4	1.4024 (19)	C11—H11A	0.9900
C3—H3	0.9500	C11—H11B	0.9900
C4—C5	1.3791 (18)	C12—C13	1.5282 (19)
C4—H4	0.9500	C12—H12A	0.9900
C5—C6	1.4026 (18)	C12—H12B	0.9900
C5—H5	0.9500	C13—C14	1.5299 (17)
C6—C7	1.4452 (17)	C13—H13A	0.9900
C7—C8	1.3690 (18)	C13—H13B	0.9900
C7—C9	1.5146 (17)	C14—H14A	0.9900
C8—C9^i^	1.5080 (17)	C14—H14B	0.9900
			
C1—N1—C8	109.12 (11)	C14—C9—C10	111.20 (10)
C1—N1—H1	124.3 (10)	C11—C10—C9	115.59 (10)
C8—N1—H1	126.3 (10)	C11—C10—H10A	108.4
N1—C1—C2	129.67 (12)	C9—C10—H10A	108.4
N1—C1—C6	107.86 (11)	C11—C10—H10B	108.4
C2—C1—C6	122.47 (12)	C9—C10—H10B	108.4
C3—C2—C1	117.50 (12)	H10A—C10—H10B	107.4
C3—C2—H2	121.2	C12—C11—C10	110.94 (11)
C1—C2—H2	121.2	C12—C11—H11A	109.5
C2—C3—C4	120.77 (12)	C10—C11—H11A	109.5
C2—C3—H3	119.6	C12—C11—H11B	109.5
C4—C3—H3	119.6	C10—C11—H11B	109.5
C5—C4—C3	121.54 (12)	H11A—C11—H11B	108.0
C5—C4—H4	119.2	C11—C12—C13	110.44 (10)
C3—C4—H4	119.2	C11—C12—H12A	109.6
C4—C5—C6	119.31 (12)	C13—C12—H12A	109.6
C4—C5—H5	120.3	C11—C12—H12B	109.6
C6—C5—H5	120.3	C13—C12—H12B	109.6
C5—C6—C1	118.40 (11)	H12A—C12—H12B	108.1
C5—C6—C7	134.98 (12)	C12—C13—C14	111.31 (10)
C1—C6—C7	106.61 (11)	C12—C13—H13A	109.4
C8—C7—C6	106.99 (11)	C14—C13—H13A	109.4
C8—C7—C9	126.21 (11)	C12—C13—H13B	109.4
C6—C7—C9	126.79 (11)	C14—C13—H13B	109.4
C7—C8—N1	109.41 (11)	H13A—C13—H13B	108.0
C7—C8—C9^i^	127.44 (12)	C13—C14—C9	115.74 (10)
N1—C8—C9^i^	123.14 (11)	C13—C14—H14A	108.3
C8^i^—C9—C7	106.34 (10)	C9—C14—H14A	108.3
C8^i^—C9—C14	111.30 (10)	C13—C14—H14B	108.3
C7—C9—C14	108.92 (10)	C9—C14—H14B	108.3
C8^i^—C9—C10	110.83 (10)	H14A—C14—H14B	107.4
C7—C9—C10	108.06 (10)		
			
C8—N1—C1—C2	−178.37 (13)	C9—C7—C8—C9^i^	0.9 (2)
C8—N1—C1—C6	0.86 (13)	C1—N1—C8—C7	−0.72 (14)
N1—C1—C2—C3	179.13 (12)	C1—N1—C8—C9^i^	179.29 (11)
C6—C1—C2—C3	−0.01 (19)	C8—C7—C9—C8^i^	−0.78 (18)
C1—C2—C3—C4	0.44 (19)	C6—C7—C9—C8^i^	−179.97 (11)
C2—C3—C4—C5	−0.2 (2)	C8—C7—C9—C14	−120.82 (13)
C3—C4—C5—C6	−0.40 (19)	C6—C7—C9—C14	59.99 (15)
C4—C5—C6—C1	0.80 (18)	C8—C7—C9—C10	118.26 (13)
C4—C5—C6—C7	−178.16 (13)	C6—C7—C9—C10	−60.93 (15)
N1—C1—C6—C5	−179.91 (11)	C8^i^—C9—C10—C11	−82.20 (13)
C2—C1—C6—C5	−0.61 (18)	C7—C9—C10—C11	161.65 (10)
N1—C1—C6—C7	−0.68 (13)	C14—C9—C10—C11	42.16 (14)
C2—C1—C6—C7	178.62 (11)	C9—C10—C11—C12	−52.32 (14)
C5—C6—C7—C8	179.29 (14)	C10—C11—C12—C13	59.53 (14)
C1—C6—C7—C8	0.25 (13)	C11—C12—C13—C14	−58.68 (14)
C5—C6—C7—C9	−1.4 (2)	C12—C13—C14—C9	50.47 (14)
C1—C6—C7—C9	179.57 (11)	C8^i^—C9—C14—C13	82.87 (13)
C6—C7—C8—N1	0.27 (14)	C7—C9—C14—C13	−160.20 (10)
C9—C7—C8—N1	−179.06 (11)	C10—C9—C14—C13	−41.22 (14)
C6—C7—C8—C9^i^	−179.73 (11)		

Symmetry codes: (i) −*x*+1, −*y*, −*z*.
